# Anterolateral, lateral, and posterior corridors to complex skull base lesions in sphenocavernous and petroclival regions: microsurgical anatomy with three-dimensional reconstructions and illustrative cases

**DOI:** 10.3389/fneur.2026.1736101

**Published:** 2026-04-07

**Authors:** Umut Tan Sevgi, Umid Sulaimanov, Taha Ş. Korkmaz, M. Hakan Şahin, Bilal Yekeler, Abdullah Keleş, Öykü Öztürk, Franco Vera Figueroa, Ufuk Erginoğlu, Abuzer Güngör, Mustafa K. Başkaya

**Affiliations:** 1 Department of Neurological Surgery, University of Wisconsin, Madison, WI, United States; 2 Department of Neurological Surgery, Health Sciences University, İzmir City Hospital, İzmir, Türkiye; 3 Department of Neurological Surgery, Onsekiz Mart University, Çanakkale, Türkiye; 4 Department of Neurological Surgery, Samsun Medicana International Hospital, Samsun, Türkiye; 5 Department of Neurological Surgery, Bayburt State Hospital, Bayburt, Türkiye; 6 Department of Neurological Surgery, Istinye University, Istanbul, Türkiye

**Keywords:** microsurgery, petroclival, photogrammetry, skull base, sphenocavernous region, three-dimensional models

## Abstract

**Objective:**

Access to complex lesions in the sphenocavernous and petroclival regions are challenging due to their deep location and proximity to critical neurovascular structures. This study aims to re-evaluate the microsurgical anatomy of the anterolateral, lateral, and posterior surgical corridors in three dimensions and present this anatomy using educational models.

**Methods:**

Pretemporal transcavernous, anterior petrosal, translabyrinthine, and far lateral approaches were performed on five cadavers. Each step was photographed, the images were processed using photogrammetry to create three-dimensional models, and 3D models were tested in an augmented reality and virtual reality environment. In addition, to correlate each anatomical corridor with its corresponding surgical application, an illustrative surgical video case was prepared for each approach.

**Results:**

Three-dimensional models were successfully created for each surgical approach in the study. The models detailed the spatial relationships of the surgical corridors in the petroclival and sphenocavernous regions and made them viewable from different angles. Tests conducted using augmented reality applications confirmed that the models could be used interactively.

**Conclusion:**

This holistic approach may be used to facilitate the selection of the appropriate corridor or combined approach based on the orientation of the lesion and its neurovascular relationships. Three-dimensional cadaveric models can be used as powerful support tools forn education and planning by enhancing spatial awareness for complex skull base surgeries.

## Introduction

Approaches to petroclival and sphenocavernous lesions constitute one of the most challenging issues of skull base surgery due to their deep location and proximity to critical neurovascular structures. Lesions in these regions frequently extend to the brainstem and cranial nerves at the time of diagnosis, making their surgical management particularly demanding (1, 2). Although minimally invasive techniques and radiosurgery offer effective treatment options for small lesions, large and complex tumors still require primary surgical resection (3–5).

New microneurosurgical techniques, intraoperative imaging methods, neurophysiological monitoring and other technological advances have led to reduced rates of surgical morbidity and mortality (6). Although endoscopic approaches have become increasingly popular in recent years, microsurgical anatomy remains a fundamental cornerstone for neurosurgeons (7–9). Particularly in complex areas such as the petroclival region, the key to surgical success depends not only on technological innovations but also on a detailed understanding of three-dimensional anatomical relationships (10, 11). Therefore, having a detailed knowledge of the relationship between surgical approaches and neurovascular 3D (three-dimensional) spatial anatomy is critical to reducing morbidity from approach selection to surgical maneuvers.

In recent years, 3D modeling, augmented reality (AR) and virtual reality (VR) applications have increasingly found their place in anatomy education and the surgical planning process (12, 13). 3D models created using photogrammetry assist in classical cadaver dissections and contribute to distance learning for those without access to laboratories, while AR and related technologies offer interactive learning opportunities (14–16). The aim of this study is to examine the detailed microsurgical anatomy of the anterolateral, lateral and posterior surgical corridors to access petroclival lesions, using the pterional approach with extradural anterior clinoidectomy and anterior petrosectomy, the translabyrinthine approach, and the far lateral approach. A second aim is to provide illustrative stepwise case demonstrations with rotatable 3D cadaveric models.

## Materials and methods

Five skull specimens obtained from routine autopsies were fixed in formalin, after which the vascular structures were stained with latex-based dyes. For demonstration purposes, pterional transcavernous, presigmoid, anterior petrosectomy, and translabyrinthine approaches were selected for access to the petroclival region via the lateral corridor. For the posterior corridor, a far lateral approach was applied to provide a broader anatomical view. These approaches were performed under a surgical microscope and photographed at each stage with a SONY A6700 mirrorless digital camera. Illustrative cases were then selected for each approach using surgical video cases prepared to demonstrate clinical applications (Supplementary Videos 1–5).

Cadaveric specimens used in this study were obtained through our institutional body donation program in accordance with ethical and legal regulations. No patient-identifiable data were included in this research. The illustrative surgical videos were selected from anonymized operative recordings prepared for educational purposes.

### Photogrammetry stage

**Figure 1:**
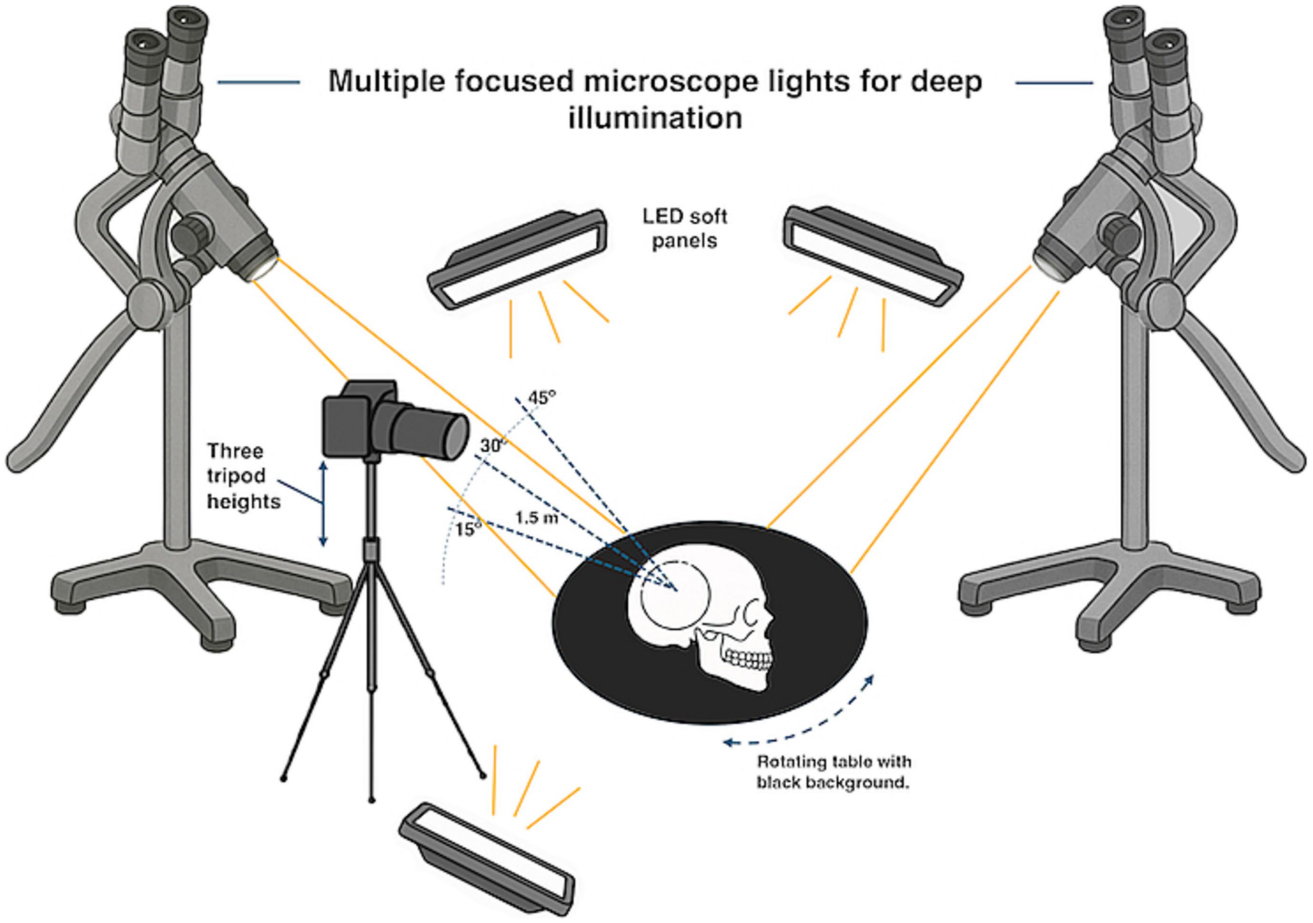
Schematic illustration of the photogrammetry stage.

3D models were created with cadaveric specimens placed on a rotatable platform covered with a black cloth and with a black background. A tripod-mounted camera was positioned ~1.5 meters away from the cadaveric specimens placed on a manually rotatable platform. Photographs were obtained at three different tripod heights for different vertical angles. At each height, the specimen was rotated 360° with photographs taken from 20 different horizontal angles for a total of 60 images for each 3D model (Figure 1).

Illumination used LED soft panels, and 2 or 3 surgical microscope light sources in order to obtain strong and focused illumination, that minimized shadow artifacts at different rotations and viewing angles. Photographic images were then processed using Agisoft Metashape photogrammetry software (Agisoft LLC) to create textured 3D surface reconstructions. The rotatable models were then exported, optimized and uploaded to Sketchfab, an online 3D visualization platform, to be available as Supplementary File 1. These models were also tested in AR and VR environments to verify their usability for interactive anatomical education.

## Results

### Pterional approach with extradural anterior clinoidectomy

**Figure 2:**
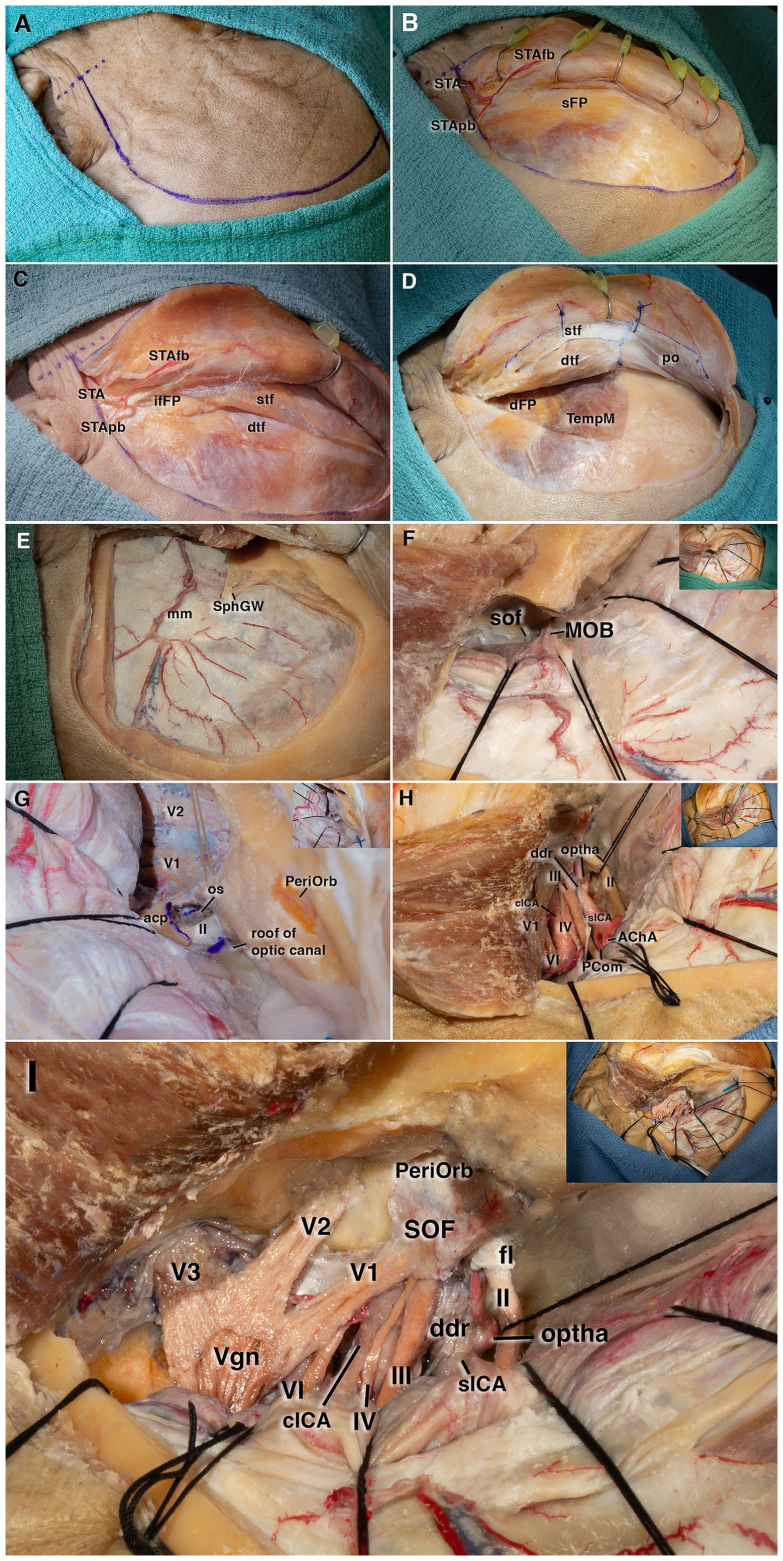
Stepwise cadaveric dissection demonstrating the pterional approach with extradural anterior clinoidectomy. **(A)** Curvilinear skin incision beginning approximately 1 cm anterior to the tragus. **(B)** Exposure of the first fat pad, the subgaleal fat pad, and the branches of the superficial temporal artery. **(C)** After elevation of the superficial layer, the interfascial fat pad (second fat pad) is visualized between the superficial and deep layers of the temporalis fascia. **(D)** Following removal of the deep temporalis fascia, the temporalis muscle and the subfascial fat pad (third fat pad) are exposed. The continuity between the superficial and deep temporalis fascia and the frontal periosteum is demonstrated. **(E)** Operative field following the pterional craniotomy. **(F)** Visualization of the meningo-orbital band during dural peeling from the sphenoid wing. **(G)** Skeletonization of the anterior clinoid process, demonstrating its three points of attachment and the reflection of the optic nerve. **(H)** After anterior clinoidectomy, exposure of the distal dural ring, clinoid segment of the internal carotid artery, anterior choroidal and posterior communicating arteries, and cranial nerves within the opened cavernous sinus dura. **(I)** Final exposure showing the optic nerve, ophthalmic artery, cavernous segment of the ICA, and related cranial nerves. AChA, anterior choroidal artery; acp, anterior clinoid process; cICA, cavernous segment of the internal carotid artery; ddr, distal dural ring; dFP, deep fat pad; dTF, deep temporal fascia; fl, falciform ligament; II–VI, cranial nerves II to VI; ifFP, interfascial fat pad; mm, middle meningeal artery; MOB, meningo-orbital band; optha, ophthalmic artery; os, optic strut; PCom, posterior communicating artery; PeriOrb, periorbita; po, periosteum; sFP, superficial fat pad; sICA, supraclinoid segment of the internal carotid artery; SOF, superior orbital fissure; SphGW, sphenoid greater wing; STA, superficial temporal artery; STAf b, frontal branch of the superficial temporal artery; STApb, parietal branch of the superficial temporal artery; stf, superficial temporal fascia; TempM, temporalis muscle; V1–V3, ophthalmic, maxillary, and mandibular divisions of the trigeminal nerve; Vgn, trigeminal (Gasserian) ganglion.

The head was rotated 20–30° contralaterally and positioned with the malar eminence as the highest point. A curvilinear skin incision was made approximately 5–10 mm anterior to the tragus, along the inferior margin of the zygomatic arch, and extended toward the midline, remaining behind the hairline whenever possible (Figure 2A). At this stage, before beginning the dissection, it is advisable to preserve the branches of the superficial temporal artery if their exposure may be required for a possible bypass (Figure 2B). During anterior mobilization of the flap, the avascular plane between the galea and the temporalis muscle–frontal periosteum was followed. The subgaleal dissection was continued until the first fat pad was reached, approximately 2.5 cm posterior to the superior orbital rim, and was then halted to avoid injury to the frontotemporal branch of the facial nerve, which courses within the subgaleal fat pad.

To demonstrate the interfascial dissection, only the superficial layer of the temporalis fascia was dissected. At this level, the second fat pad, known as the interfascial fat pad, was encountered (Figure 2C). Subsequently, the deep layer of the temporalis fascia was incised and dissected together with the periosteum in a subperiosteal plane over the muscle. Finally, the third fat pad, the subfascial fat pad, was identified over the temporalis muscle (Figure 2D). The continuity of the superficial and deep layers of the temporalis fascia with the frontal periosteum was demonstrated. A thin myofascial cuff was left on the bone, and the temporalis muscle was incised and reflected retrogradely as described by Oikawa et al. (17).

Three burr holes were placed: the first over the keyhole, the second posterior to the superior temporal line, and the third on the squamous part of the temporal bone near the superior aspect of the zygomatic root, followed by a craniotomy. The dural and the greater wing of the sphenoid bone were exposed (Figure 2E). After the dura was carefully peeled from the sphenoid bone, the sphenoid wing was drilled until the anterior and middle cranial fossae formed almost a continuous flat plane. Then, the meningo-orbital band entering the orbit from the superior portion of the superior orbital fissure (SOF) was identified and divided (Figure 2F). As the dura propria was elevated from the orbital roof toward the middle cranial fossa, the margins of the SOF were dissected, exposing the ophthalmic (V1) and maxillary nerves (V2). At this stage, the anterior clinoid process (ACP) was exposed, and its margins were defined (Figure 2G). Following a small orbitotomy, the SOF was decompressed using continuous irrigation and a powered drill, and optic unroofing was performed. The three attachment points of the ACP, the sphenoid wing, optic roof, and optic strut, were drilled, freeing the ACP, which was then detached from the lateral wall of the optic canal to complete the clinoidectomy. After removal of the ACP, the internal carotid artery (ICA) coursing beneath the membrane at the base of the anteromedial triangle was exposed from the proximal to the distal dural ring (Figure 2H).

### Exposing the cavernous sinus

Within the cavernous sinus (CVS) region, peeling the dura propria from the lateral wall of the CVC and dissecting along the cleavage plane exposed the oculomotor, trochlear, ophthalmic, and maxillary nerves. The outer dural layer was retracted with sutures, then incised posteriorly along the lateral margin of the CVC over the maxillary nerve and extended toward the mandibular nerve (V3). The middle meningeal artery (MMA) was then cut to enhance exposure. After suction of the venous blood from the cavernous sinus, the neural anatomy became more distinct. In the lateral wall, the oculomotor, trochlear, ophthalmic, and maxillary nerves were identified. Medially, the lateral wall of the sella turcica was visualized, and posteriorly, the CVC was seen merging with the basilar venous plexus. Posterior to the bony margin of the SOF, the trochlear nerve was observed ascending along the lateral surface of the oculomotor nerve and crossing over it. As it continued anteriorly, the trochlear nerve was identified entering the fissure above and lateral to the oculomotor nerve. The abducens nerve, as it advanced anteriorly within the CVS, was observed to descend slightly and course more medially before entering the SOF (Figure 2I).

### Anterior transpetrosal approach

After performing a pterional craniotomy, the dura was dissected anteriorly toward the region of the foramen spinosum and foramen ovale. The MMA was coagulated and divided close to the temporal dura, rather than at the foramen, to avoid injury to the petrosal branch of the MMA, which supplies the geniculate ganglion and the horizontal segment of the facial nerve. The temporobasal dura was then mobilized and carefully dissected from the Gasserian ganglion. At the posteromedial margin of the V3, the greater superficial petrosal nerve (GSPN) was identified and skeletonized in a posterior-to-anterior fashion to ensure its preservation and avoid inadvertent injury.

**Figure 3:**
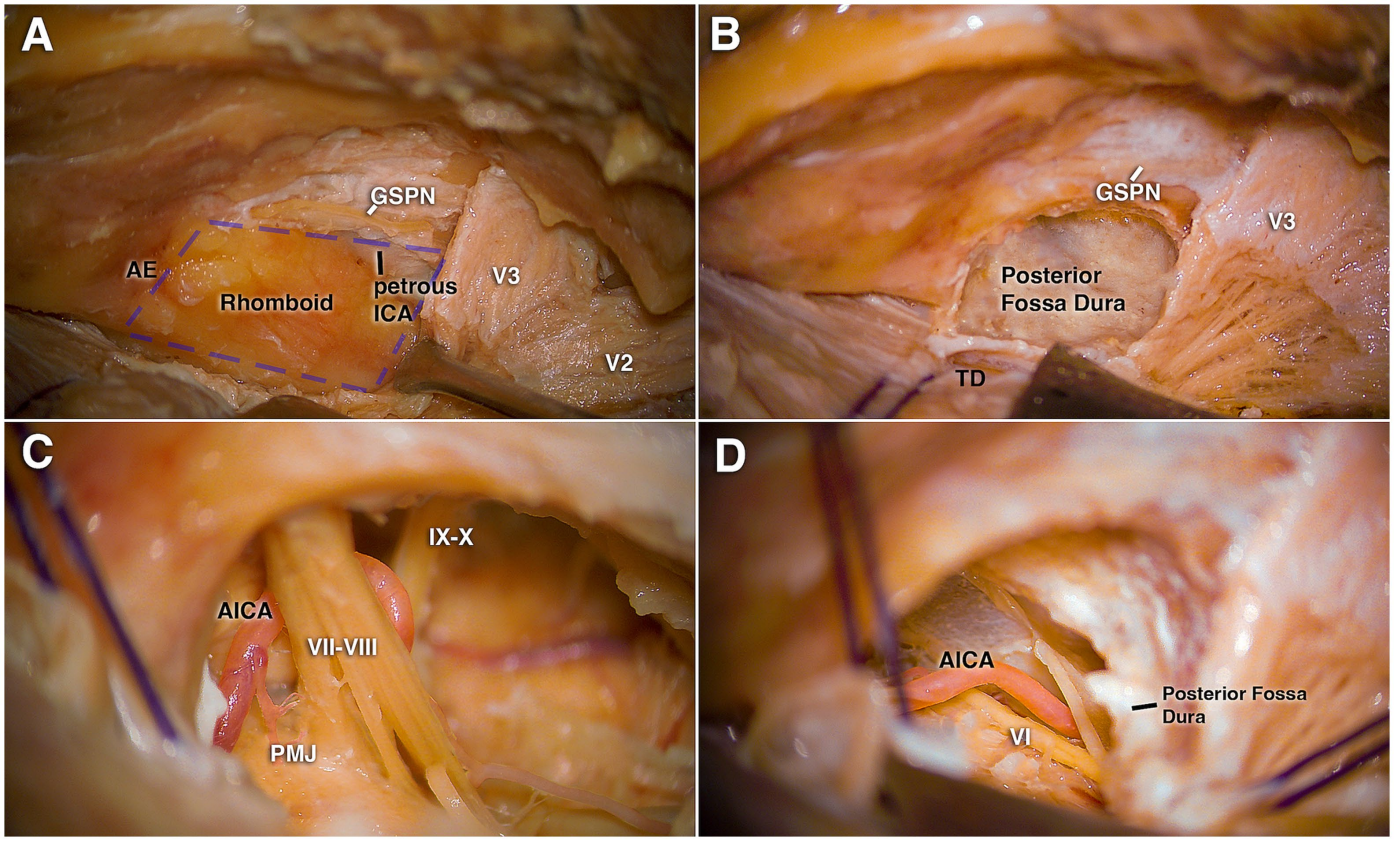
Demonstrating the anterior transpetrosal (Kawase) approach following pterional craniotomy. **(A)** After elevation of the temporal dura from the middle fossa, the greater superficial petrosal nerve (GSPN), trigeminal nerve, arcuate eminence, and petrous ridge forming the boundaries of the rhomboid are identified. **(B)** Exposure of the posterior fossa dura following drilling of the rhomboid fossa. **(C)** After dural opening, visualization of the seventh and eighth cranial nerve complex together with the anterior inferior cerebellar artery (AICA). **(D)** A more ventral view showing the sixth cranial nerve at the pontine level. AICA, anterior inferior cerebellar artery; AE, arcuate eminence; GSPN, greater superficial petrosal nerve; ICA, internal carotid artery; IX–X, glossopharyngeal and vagus nerves; TD, temporal dura; PMJ, pontomedullary junction; V2, maxillary division of the trigeminal nerve; V3, mandibular division of the trigeminal nerve; VI, abducens nerve; VII–VIII, facial and vestibulocochlear nerves.

The dura was peeled from the petrous surface, thereby exposing the arcuate eminence, the petrous ridge, and the course of the GSPN. Retraction sutures were applied to elevate the dura and temporal lobe overlying the Gasserian ganglion, and an extradural retractor blade was secured to the dura covering the petrous ridge (Figure 3A).

Drilling was initiated at the petrous ridge and progressively expanded anteriorly toward the trigeminal ganglion, laterally along the GSPN, and dorsally toward the arcuate eminence. A thin shell of bone was preserved over the petrous ICA, which was skeletonized with awareness that its osseous covering may be incomplete. Drilling was then carried posteriorly to expose the posterior fossa dura (Figure 3B). At this stage, the dura over the IAC was exposed, and bone removal was extended caudally in the direction of the jugular foramen, without completing full exposure of the foramen.

The key step of the anterior petrosectomy was the removal of the bone between the carotid canal and the area leading to the IAC, thereby exposing Kawase’s rhomboid. This safe drilling zone is bounded laterally by the GSPN, medially by the petrous ridge, posteriorly by the arcuate eminence, and anteriorly by the trigeminal ganglion and V3. Once drilling of Kawase’s triangle was completed, the dura over the middle and posterior fossae was opened and the superior petrosal sinus was ligated and divided, creating communication between the middle and posterior fossa (Figures 3C,D).

### Translabyrinthine approach

**Figure 4:**
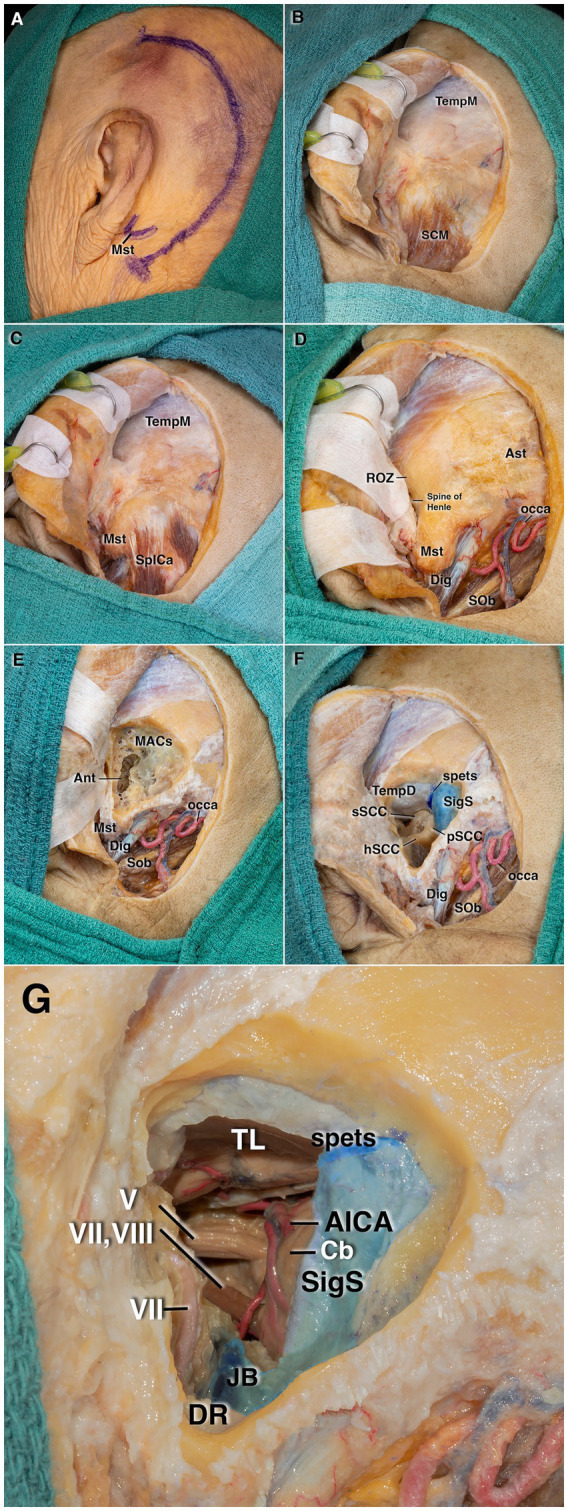
Stepwise cadaveric dissection demonstrating the translabyrinthine approach. **(A)** C-shaped retroauricular skin incision. **(B)** After anterior reflection of the myocutaneous flap, exposure of the superficial muscle group. **(C)** Elevation of the sternocleidomastoid muscle revealing the splenius capitis. **(D)** After removal of the splenius capitis, the occipital artery, digastric muscle, and superior oblique muscle are visualized. The boundaries of the drilling area are identified by the root of the zygoma, mastoid tip, and asterion. **(E)** Initial mastoid drilling demonstrates the mastoid air cells and the mastoid antrum at their point of coalescence. **(F)** After identification of the digastric ridge, incus, and the facial nerve trajectory, retrofacial drilling exposes the semicircular canals. The overlying temporal dura is also demonstrated superiorly. **(G)** Final exposure showing the facial–vestibulocochlear nerve complex and the anterior inferior cerebellar artery (AICA), with a ventral view toward the cerebellopontine angle (CPA). The temporal dura has been opened for demonstration purposes. AICA, anterior inferior cerebellar artery; Ant, antrum; Ast, asterion; Cb, cerebellum; Dig, digastric muscle; DR, digastric ridge; hSCC, horizontal semicircular canal; JB, jugular bulb; MACs, mastoid air cells; Mst, mastoid; occa, occipital artery; pSCC, posterior semicircular canal; ROZ, retrosigmoid opening zone; SCM, sternocleidomastoid muscle; SigS, sigmoid sinus; Sob, superior oblique muscle; SpICa, splenius capitis muscle; spets, superior petrosal sinus; sSCC, superior semicircular canal; TempD, temporal dura; TempM, temporalis muscle; TL, temporal lobe; V, trigeminal nerve; VII, facial nerve; VII–VIII, facial and vestibulocochlear nerves.

The head was rotated 60–80° to the contralateral side. A wide C-shaped incision was made beginning approximately 4–5 cm above the external auditory canal and extending inferiorly to about 2 cm below the mastoid tip, allowing visualization of the surrounding muscular structures (Figure 4A). The temporalis muscle was identified superiorly, and the sternocleidomastoid muscle was identified more superficially in the inferior portion (Figure 4B). During surgery, fascia from the temporalis muscle may be harvested for use as a dural graft. The sternocleidomastoid muscle, coursing antero-inferiorly, was dissected from the superior nuchal line, after which the prevertebral cervical fascia was elevated, exposing the occipital artery. Beneath it, the superior oblique and digastric muscles were identified, completing the muscular dissection. The mastoid tip, external auditory canal, spine of Henle, asterion, and retrosigmoid region were then fully exposed (Figures 4C,D). The asterion, the posterior corner of the root of the zygoma (corresponding approximately to the level of the middle fossa floor and the transverse sinus), and the mastoid tip were used as the vertices of a surgical triangle, with the spine of Henle serving as the anterior boundary. Drilling was then performed within this triangle using a powered drill.

The sigmoid sinus was skeletonized, and the mastoid air cells were visualized. Although the asterion generally corresponds to the posterior margin of the sigmoid sinus, it should not be considered a reliable landmark, as the exact boundaries of the sinus become apparent progressively during mastoidectomy. In this region, the mastoid antrum, a large cavity formed by the coalescence of mastoid air cells, was identified (Figure 4E). Advancing from the antrum toward the middle ear, the short process of the incus was visualized. Anterior to the sigmoid sinus, at the junction with the mastoid tip, a compact bony ridge known as the digastric ridge was identified on the inner surface. After identification of the mastoid antrum, drilling was continued posteriorly, exposing the presigmoid dura, middle fossa dura and the sinodural angle formed between the sigmoid sinus and the middle fossa. The bony layer covering the sigmoid sinus was followed from the transverse sinus to the mastoid tip. The presigmoid mastoid air cells located deep to the digastric ridge were drilled out, exposing the jugular bulb. The incus and the digastric ridge serve as important landmarks for identifying the trajectory of the facial nerve within the mastoid. After defining these structures, retrofacial drilling was performed, exposing the bony labyrinth (Figure 4F). As the posterior semicircular canal was separated from the posterior fossa dura, the vestibular aqueduct—a dural fold extending inferior to the canal—was identified and divided, and the dissection was continued toward the IAC.

Drilling was continued parallel to an imaginary line connecting the ampullae of the superior and posterior semicircular canals. After completing the labyrinthectomy, the thin bony layer remaining over the dura was removed. The dura was opened parallel to and anterior to the sigmoid sinus. The temporal dura was resected, exposing the temporal lobe. The anterior inferior cerebellar artery (AICA), cerebellum, and the trigeminal nerve were exposed in the cerebellopontine angle (Figure 4G).

### Far lateral approach

**Figure 5:**
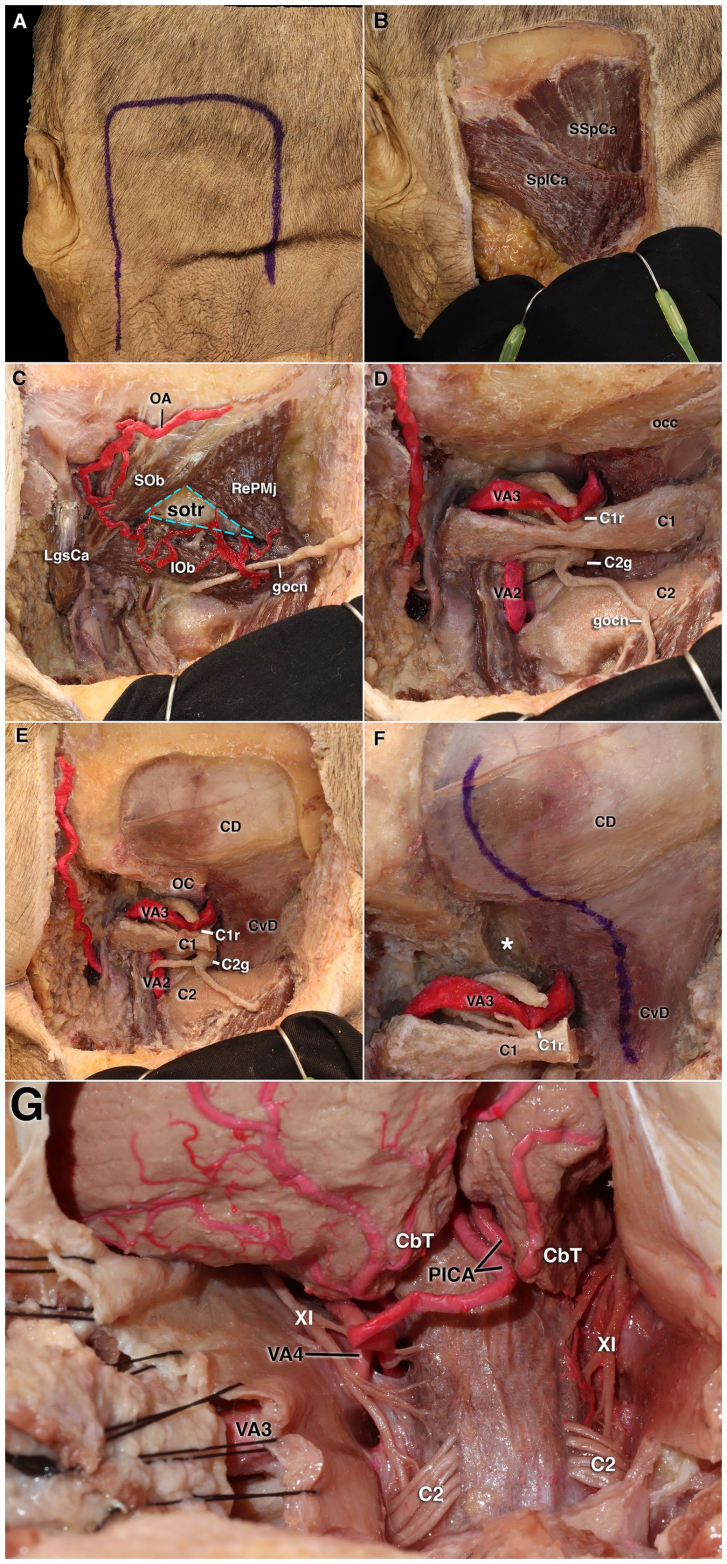
Stepwise cadaveric dissection demonstrating the far-lateral approach. **(A)** Horseshoe-shaped skin incision. **(B)** Exposure of the splenius capitis and semispinalis capitis muscles. **(C)** Revealing the suboccipital triangle. **(D)** Visualization of the C1 posterior arch and vertebral artery. **(E)** After suboccipital craniectomy and removal of the C1 posterior arch, exposure of the cerebellomedullary cistern. **(F)** S-shaped dural incision. **(G)** Final exposure demonstrating access to the lower clivus and petroclival region, with visualization of the lower cranial nerves. C1, first cervical vertebra; C1r, C1 root; C2, second cervical vertebra; C2g, C2 ganglion; CbT, cerebellar tonsil; CD, cerebellar dura; CvD, cervical dura; gocn, greater occipital nerve; IO, inferior oblique muscle; LgsCa, longissimus capitis muscle; OA, occipital artery; OC, occipital condyle; occ, occipital bone; PICA, posterior inferior cerebellar artery; RePMj, rectus capitis posterior major muscle; SOb, superior oblique muscle; SSpCa, semispinalis capitis muscle; SPlCa, splenius capitis muscle; sotr, suboccipital triangle region; VA2–VA4, second to fourth segments of the vertebral artery; VA3, third segment of the vertebral artery; XI, accessory nerve.

The medial limb of the incision began approximately 5–6 cm below the inion, extended upward toward it, and then followed the superior nuchal line (Figure 5A). From there, it curved downward anterior to the posterior border of the sternocleidomastoid muscle, reaching the level of the transverse process of the C1 vertebra, forming a horseshoe-shaped incision. After the flap was reflected, the most superficial layer revealed the sternocleidomastoid and splenius capitis muscles laterally, and the semispinalis capitis muscle medially (Figure 5B). The longissimus capitis muscle was identified laterally. After removal of this muscle layer, the suboccipital triangle, formed by the superior oblique muscle superolaterally, the inferior oblique muscle inferolaterally, and the rectus capitis major muscle superomedially, was identified (Figure 5C). The triangle contained dense fatty and fibrous tissue. At this stage, the posterior arch of the atlas could be easily palpated. Removal of these muscular structures made it possible to identify the C1 nerve and the vertebral artery (VA) coursing within the groove on the posterior arch of the atlas. The venous plexus surrounding the VA was then cleared (Figure 5D).

Subsequent removal of the remaining muscular structures made the C1 and C2 ganglia, the occipital bone, and the vertebral artery clearly visible. The VA was observed to have muscular branches that passed medially behind the atlanto-occipital joint and over the posterior arch of C1, then turning upward and forward to pierce the dura and enter the intracranial space. Using the asterion, located near the junction where the transverse sinus turns into the sigmoid sinus, as the superolateral landmark, a lateral suboccipital craniectomy was performed. The posterior arch of the C1 ganglia was then removed, exposing the atlanto-occipital joint and the occipital condyle (Figure 5E). Drilling the condyle further expanded the exposure (Figure 5F).

**Figure 6:**
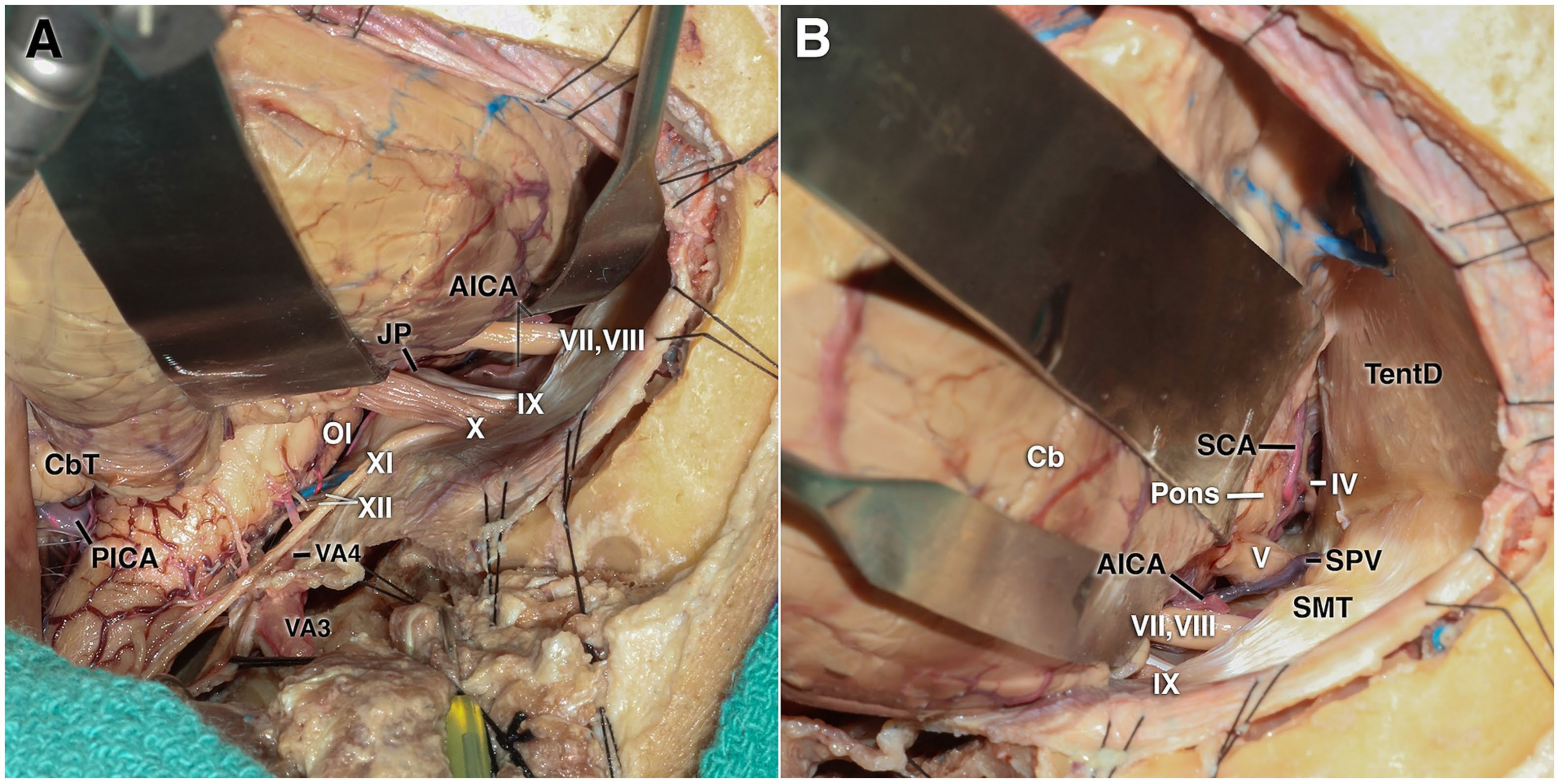
Cadaveric dissection demonstrating the retrosigmoid corridor. **(A)** After gentle cerebellar retraction, exposure of the lower cranial nerves at the cerebellomedullary cistern. **(B)** A more superior view showing the tentorial dura, fourth cranial nerve, superior petrosal vein, and trigeminal nerve. AICA, anterior inferior cerebellar artery; Cb, cerebellum; CbT, cerebellar tonsil; IV, trochlear nerve; IX–XII, glossopharyngeal, vagus, accessory, and hypoglossal nerves; JP, jugular process; OI, olive; PICA, posterior inferior cerebellar artery; SCA, superior cerebellar artery; SMT, supra meatal tubercule; SPV, superior petrosal vein; TentD, tentorial dura; VA3–VA4, third and fourth segments of the vertebral artery; V, trigeminal nerve; VII–VIII, facial and vestibulocochlear nerves; X, vagus nerve; XI, accessory nerve; XII, hypoglossal nerve.

The dura was opened with an S-shaped incision, beginning posterior to the sigmoid sinus and extending behind the VA down to the level of C1. At this level, care was taken to preserve the marginal sinus encircling the foramen magnum and the posterior meningeal artery. Opening the dura exposed the intradural segment of the VA. The cerebellar tonsils, the tonsillomedullary segment of the posterior inferior cerebellar artery, and the roots of the glossopharyngeal, vagus, and accessory nerves were also identified (Figure 5G). Proceeding superiorly along the lateral surface of the cerebellum demonstrated the retrosigmoid approach, revealing the course of the seventh and eighth cranial nerves, their relationship with the AICA, and the jugular process (Figure 6A). Advancing further superiorly exposed the tentorial dura, the superior cerebellar artery, the fourth and fifth cranial nerves, the superior petrosal vein, and the suprameatal tubercle (Figure 6B).

### 3D models

**Figure 7:**
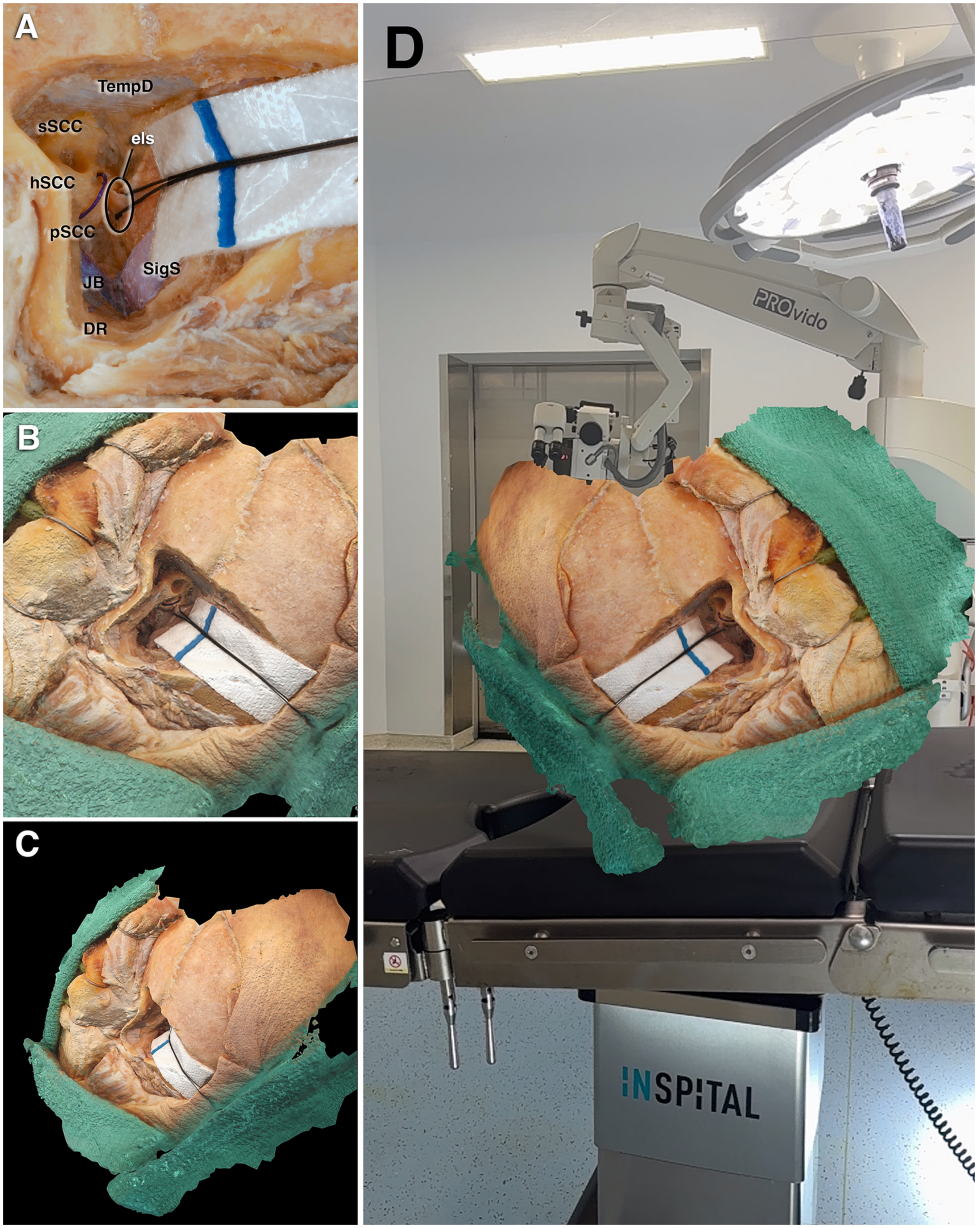
Cadaveric dissection and virtual 3D modeling. **(A)** In another specimen, demonstration of the endolymphatic sac posterior to the semicircular canals, retracted with a suture after completion of the transmastoid dissection. **(B–C)** Virtual 3D model of the same specimen viewed from different angles; the model can be freely rotated and scaled to any desired size, allowing detailed and dynamic visualization of the surgical anatomy. **(D)** Visualization of the model in augmented reality, enabling interactive examination within the operating room environment. DR, digastric ridge; ELS, endolymphatic sac; hSCC, horizontal semicircular canal; JB, jugular bulb; pSCC, posterior semicircular canal; sSCC, superior semicircular canal; SigS, sigmoid sinus; TempD, temporal dura.

A total of 26 3D models were created to show the surgical steps for the 3 different approaches (Anterior Transpetrosal. Translabyrinthine, and Far Lateral) with various surgical perspectives that can be viewed with 3D rotation and size scaling. Models were exported in .glb and .usdz formats to enable viewing with smartphones, other mobile devices, and VR headsets (Figures 7A–D). Model files ranged from 400 MB to 1.3 GB, and with each rendered in 2 ± 0.5 h, depending on texture complexity and lighting. The 3D models are freely accessible in Supplementary File 1.

## Discussion

Surgical approaches to the petroclival and sphenocavernous regions constitute one of the most challenging skull base areas. This stems from deep-seated lesions, complex neurovascular relationships, thick bony barriers and oftentimes inadequate knowledge of microneurosurgical anatomy combined with the limited sensitivity of preoperative radiology (18–21). These factors limits both the surgeon’s choice of approach and intraoperative maneuvering space. Our objective here is to reassess conventional surgical approaches with a 3D perspective and demonstrate that the individually defined surgical windows actually constitute a continuous and complementary anatomical corridor.

The anterolateral, lateral, and posterior corridors provide access to the petroclival and sphenocavernous regions from different directions but within a unified anatomical framework. When examined sequentially in the same cadaveric series, these approaches can be understood as converging within a single 3D plane. Thus, techniques traditionally taught as separate procedures may, in fact, represent complementary perspectives of one complex anatomical continuum, extending from the ACP to the sella, continuing to the clivus, and reaching inferiorly to the foramen magnum. Understanding this region through the pterional, anterior petrosal, presigmoid, and retrosigmoid paradigms may also demonstrate how lesions extending across adjacent compartments can be approached more safely and effectively through stepwise or combined routes with minimal tissue disruption.

To better understand access to the petroclival region, the clivus can be divided anatomically into four quadrants (22). The upper quadrant extends from the dorsum sellae to the petrous apex. The second quadrant encompasses the region between the petrous apex and the IAC. The third quadrant includes the portion from the IAC to the jugular tubercle. The lower quadrant extends from the jugular tubercle to the anterior margin of the foramen magnum. Different levels of the clivus can be targeted through various complementary surgical approaches. The upper quadrant of the clivus can be accessed via pterional transsylvian, pretemporal transcavernous, subtemporal, and anterior petrosal (Kawase) approaches. The second quarter can be visualized through a combination of the anterior petrosal and posterior petrosal approaches with subtemporal extensions. The third quarter is revealed through a posterior petrosal–subtemporal combination as well as a far lateral approach. The lower quarter is targeted through far lateral and juxtacondylar approaches. However, considering these regions as a continuous anatomical space rather than isolated areas allows the surgeon to use or combine anterolateral, lateral, and posterior routes more logically and safely during surgical planning (1, 23).

### Surgical approaches

The pretemporal transcavernous approach provides extradural access to basilar tip aneurysms, retrochiasmatic and intraventricular lesions, selected petroclival tumors, as well as the pituitary fossa and CVS region (24). Although the surgical field is relatively narrow, this approach allows exposure of the ventral brainstem by reducing direct parenchymal retraction. When deeper or inferior extensions need to be targeted, the surgical corridor can be widened by performing anterior and posterior clinoidectomy (10, 24–26).

Removal of the ACP significantly increases the medial angle of the approach and offers several advantages (27). Opening the clinoidal space allows early proximal control at the level of the clinoidal ICA. Exposure of the oculomotor nerve facilitates its mobilization and guides the direction of intradural dissection; furthermore, opening the clinoid space allows clear visualization of the ophthalmic and posterior communicating arteries, as well as the posterior clinoid process, which may need to be removed. The length, course, and thickness of the posterior communicating artery may limit access to the ventral brainstem and its perforators. In this case, coagulating and dividing perforator free segments of the artery allows for a wider exposure of the interpeduncular fossa (24, 28).

The anterior petrosal approach involves drilling through the rhomboid, Meckel’s cavity, and the ventral brainstem above the IAC, providing access to the petroclival junction (29, 30). This extradural approach reduces brain retraction and the risk of Labbé vein injury (11, 31). Furthermore, It may be used independently, such as for pontine cavernomas, or extended through transtentorial or presigmoid routes to provide broader exposure (11, 32).

The posterior petrosal approaches extend through the mastoid and petrous portions of the temporal bone, providing access to the central skull base and the anterolateral aspect of the brainstem (33, 34). These are primarily indicated for petroclival lesions that may be difficult to reach using conventional routes such as the retrosigmoid approach (35, 36). The posterior petrosal approaches include three principal variants, retrolabyrinthine, translabyrinthine, and transcochlear, with several possible modifications and combinations. The fundamental concept underlying all these approaches is to achieve safe access to the ventral aspect of the brainstem while minimizing retraction or traction on the cranial nerves and brainstem. The retrolabyrinthine approach involves removal of the bone located between the sigmoid sinus posteriorly and the semicircular canals anteriorly. It preserves hearing; however, when used alone, it provides a relatively limited surgical corridor (37–39). To enhance exposure, it is often combined with a middle fossa craniotomy and transection of the superior petrosal sinus (40–43). Preoperative radiological evaluation should include a detailed assessment of the venous drainage of the vein of Labbé, to determine whether it drains into the superior petrosal sinus, tentorium, or transverse sinus (44). The retrolabyrinthine approach is more suitable for patients with preserved hearing. When a tentorial incision is required, it should be made anteriorly to avoid injury to the trochlear nerve.

The translabyrinthine approach represents one step forward, providing a more ventral route by removing the semicircular canals and vestibule (45–48). It allows access to the IAC, the canalicular segments of cranial nerves VII and VIII, the cisternal segment of the trigeminal nerve, and the posterior half of the incisural space. This approach is preferred in patients with non-serviceable hearing or in borderline cases where postoperative hearing loss is anticipated, as drilling of the vestibular structures inevitably sacrifices hearing (46, 49). It provides a direct route to the cerebellopontine angle and IAC, allowing easier access to the most superior portion of the facial nerve without excessive cerebellar retraction. This facilitates early identification, decompression, and mobilization of the facial nerve at the lateral end of the IAC. However, for tumors located far anteriorly or those extending caudally to the lower clivus with a wide inferior brainstem attachment, surgical access may become challenging, particularly when a high jugular bulb limits the inferior exposure (49–52). This approach, being almost nearly epidural, is a suitable option for vestibular schwannomas in patients with non-serviceable hearing. The transcochlear approach provides an even more ventral trajectory; however, it carries an additional risk of facial weakness along with hearing loss, as it requires transposition of the facial nerve (53–55).

The combined petrosal approach, combining the anterior petrosal and retrolabyrinthine presigmoid routes, can provide wide and multidirectional exposure of the petroclival junction, middle clivus, Meckel’s cave, and posterior CVS while preserving hearing and facial nerve function (56–59). In addition, the anterior component allows early devascularization of tumors such as meningiomas, facilitating resection and providing adequate working space even in the presence of a dominant sigmoid sinus or large bridging veins (21, 23, 60). Moreover, posterior petrosal approaches can be combined with the retrosigmoid or far-lateral routes to extend exposure toward the cerebellomedullary cistern (61–63).

The retrosigmoid and far-lateral approaches provide access to more inferior extensions and are generally more familiar to neurosurgeons. Although the retrosigmoid approach is faster and technically more comfortable, it offers only limited exposure of the anterior cerebellopontine angle, ventral brainstem, and petroclival region (64–66). Therefore, depending on the tumor pathology, this approach may be restrictive for lesions extending anterior to the trigeminal nerve or the facial nerve. In addition, prolonged cerebellar retraction may cause injury to the cerebellar tissue (67). Nevertheless, with adjuncts such as tentorial incision or suprameatal drilling, the retrosigmoid approach can be effectively used in selected petroclival tumors (68, 69). Among these, the retrosigmoid approach remains one of the most versatile and widely adopted techniques for posterior fossa surgery. The far-lateral approach provides a similar trajectory but is particularly useful for more inferior lesions, including those extending anterior to the foramen magnum and for vertebrobasilar aneurysms. During exposure, the most critical step in the opening of the lateral suboccipital region is the identification and preservation of the VA, which must be meticulously protected from injury. Drilling of the occipital condyle, while protecting the hypoglossal canal, can enhance exposure; however, the potential risk of craniocervical instability should always be considered.

### Three-dimensional reconstruction, educational value, and future directions

In the present study, 3D reconstructions were generated using photogrammetry, a technique that creates virtual 3D models by digitally merging photographs taken from multiple angles through specialized software. Its use in neurosurgery has been established in recent years and has increasingly emerged as a valuable tool in anatomical education and neurosurgical training (12, 13, 15, 70, 71).

Smartphone-based photogrammetry has yielded successful results in rapidly generating and sharing 3D models (12, 13). Models generated from photographs taken with DSLR cameras, although produced through a slower process, have achieved higher image quality and anatomical fidelity (71, 72). With wide external fixed lighting, moving the camera closer for detailed shots further darkens already shadowed areas. Conversely, providing strong external illumination sufficient to brighten deep regions causes overexposure and glare in surrounding, well-lit areas. In studies utilizing DSLR-based photogrammetry, a fixed lighting setup and an automated rotating turntable have typically been employed (12, 13, 71). In our study, in addition to standard external light sources, each photograph was individually illuminated using OPMI microscope lights. Owing to the narrow and focused beam of the microscope light, this prevents the camera from casting shadows into the deep surgical corridor, thereby minimizing shadow-related artifacts. These lights provided strong, precise illumination sufficient for deep surgical fields while avoiding unnecessary exposure of areas outside the corridor. Using multiple surgical microscopes, focused light could be directed from multiple angles in the surgical corridors, allowing structures to be clearly visualized without shadowing thereby enabling realistic 3D reconstructions. Conceptually, the most appropriate method for this process would be to capture photographs directly through the surgical microscope under optimal illumination to generate the models. However, the image quality obtained from microscope-mounted cameras remains inferior to that of DSLR systems (73). Another challenge arises from the shorter focal distance of microscope-mounted cameras compared with DSLR systems; during close-range imaging of deep structures, regions outside the focal plane appear blurred, reducing overall image sharpness and depth fidelity. Ballesteros-Herrera et al. generated high-quality models by applying focus stacking to photographs taken at varying focal distances using advanced imaging equipment (74). This technique effectively enhanced depth resolution by integrating multiple focused layers into a single composite image. However, the major limitation in modeling deep and narrow surgical approaches remains the restricted and poorly illuminated working space. Although the use of mobile microscope lights, as in our study, partially mitigates this issue by improving illumination within narrow corridors, complete resolution would likely require imaging systems designed specifically for such environments—ideally with optical characteristics similar to those of surgical microscopes (Perhaps in future studies, a system that combines the high image quality of DSLR cameras with the focused illumination of a surgical microscope could overcome these limitations, enabling high-resolution reconstructions through focus stacking even in deep and narrow anatomical corridors.) Successful implementation of the method we used depends on the availability of a laboratory equipped with multiple surgical microscopes or light sources capable of delivering comparable intensity and focal precision. This likely explains why prior photogrammetry-based studies that aimed to depict deep structures such as the cavernous sinus, have often required removal of surrounding tissues to enhance illumination (70, 75). In many reports, reconstructions have been limited to the craniotomy level, without successful demonstration of full surgical corridors (76, 77).

Anatomical education, which began with traditional hand-drawn illustrations, has evolved through the use of digital renderings and, more recently, 3D photographic models (71, 78, 79). The advantage of these 3D models lies in their ability to be freely rotated, examined from any angle, and facile access through digital platforms. While 3D models generated from digital drawings or radiological segmentations are useful, they lack the natural texture and tissue characteristics of real anatomy. In this regard, 3D cadaveric models can provide the similar resemblance to tissue, offering a more realistic representation of anatomical structures (13, 80, 81). However, cadaveric dissection-based learning remains irreplaceable due to its tactile feedback and the direct sensory experience it provides in exploring anatomical relationships. Nevertheless, in low-income countries or regions with limited access to anatomical laboratories, such 3D models may serve as a valuable alternative, thus functioning as effective educational tools and as supplementary resources for knowledge reinforcement and skill retention (12, 13, 82–84).

AR enables the models to be examined within the real environment, allowing the surgeon to appreciate how changes in head position and operative posture alter the perceived spatial relationships of the corridor. VR, in contrast, provides complete immersion, where depth and orientation can be explored more intuitively. Experiencing these perspectives may improve spatial orientation, support confidence in approach selection, and facilitate teaching of complex skull base anatomy (12–15, 85).

To the best of our knowledge, this is the first study that demonstrates surgical corridors to the sphenocavernous and petroclival regions via step-by-step 3D models with the support as illustrative clinical cases. The findings of this study suggest that access to the petroclival and sphenocavernous regions can be better understood as arising from complementary axes within a single 3D anatomical plane rather than entirely separate windows. This integrative perspective may assist surgeons in selecting stepwise or combined approaches based on the direction of lesion extension. In the future, integrating cadaveric models with radiological segmentation, intraoperative navigation, and neurophysiological mapping data may provide a foundation for real-time, patient-specific surgical corridor planning.

## Conclusion

Anterolateral, lateral, and posterior corridors in the petroclival and sphenocavernous regions should be regarded not as single, fixed routes but as complementary, multi-axial surgical windows. Appropriate corridor selection may assist the surgeon in tailoring the approach according to the lesion’s orientation and its neurovascular relationships, with the primary goal of brainstem decompression followed by safe resection. 3D cadaveric models and AR applications may further enhance spatial orientation and depth perception, thereby reinforcing microsurgical anatomy and supporting a safer, more intuitive, and teachable level of planning for complex skull base surgery.

## Data Availability

The datasets presented in this study can be found in online repositories. The names of the repository/repositories and accession number(s) can be found in the article/Supplementary material.

## Supplementary material

SUPPLEMENTARY VIDEO 1 Surgical demonstration of resection of the trigeminal schwannoma via the frontotemporal transcavernous approach (https://doi.org/10.6084/m9.figshare.30488228.v1).

SUPPLEMENTARY VIDEO 2 Surgical demonstration of resection of the petroclival meningioma via the anterior petrosal (Kawase) approach (https://doi.org/10.6084/m9.figshare.30488330.v1).

SUPPLEMENTARY VIDEO 3 Surgical demonstration of resection of the vestibular schwannoma via the translabyrinthine approach (https://doi.org/10.6084/m9.figshare.30488378.v1).

SUPPLEMENTARY VIDEO 4 Surgical demonstration of resection of the vestibular schwannoma via the retrosigmoid approach (https://doi.org/10.6084/m9.figshare.30488423.v2).

SUPPLEMENTARY VIDEO 5 Surgical demonstration of resection of the foramen magnum meningioma via the far lateral approach (https://doi.org/10.6084/m9.figshare.30488462.v1).

SUPPLEMENTARY FILE 1 3D models of Cadaveric dissections. 3D models are freely accessible at: https://sketchfab.com/Istanbulmicroneurosurgery/collections/mkb-collection-e5714ae5b66347058c429e30a5d09282.
